# Chinese herbal decoction (Danggui Buxue Tang) supplementation augments physical performance and facilitates physiological adaptations in swimming rats

**DOI:** 10.1080/13880209.2020.1774622

**Published:** 2020-06-13

**Authors:** William Chih-Wei Chang, Ching-Chi Yen, Chao-Pei Cheng, Yu-Tse Wu, Mei-Chich Hsu

**Affiliations:** a School of Pharmacy, Kaohsiung Medical University, Kaohsiung, Taiwan; b Drug Development and Value Creation Research Center, Kaohsiung Medical University, Kaohsiung, Taiwan; c Department of Medical Research, Kaohsiung Medical University Hospital, Kaohsiung, Taiwan; d Department of Sports Medicine, Kaohsiung Medical University, Kaohsiung, Taiwan; e Substance and Behavior Addiction Research Center, Kaohsiung Medical University, Kaohsiung, Taiwan

**Keywords:** Traditional Chinese medicine, sports nutrition, ergogenic aid, nutritional supplement, exercise, *Angelica sinensis*

## Abstract

**Context:**

Danggui Buxue Tang (DBT), one of the popular Danggui (DG) decoctions, has traditionally been used to nourish ‘qi’ (vital energy) and enrich ‘blood’ (body circulation). DBT may possess performance-enhancing effects.

**Objective:**

This work determines whether DBT can improve physical capacity and alter energy expenditure under exercise training.

**Materials and methods:**

Forty male Wistar rats were assigned to four groups: sedentary (SE), exercise training (ET), ET supplemented with 0.3 g/kg rat/d DG extract, and ET supplemented with 1.8 g/kg rat/d DBT extract. The supplementations were administered via oral gavage. During the 21-day treatment period, the exercised groups were subjected to a protocol of swimming training with a gradually increased load. Physical performance evaluation was assessed using the forelimb grip strength test and an exhaustive swimming test. Muscle glycogen contents and exercise-related biochemical parameters were analysed.

**Results:**

Both herbal supplementations remarkably increased the grip strength (DG by 49.7% and DBT by 85.7%) and prolonged the swimming time (DG by 48.4% and DBT by 72.7%) compared with SE. DBT spared a certain amount of glycogen in the muscle cells under exercise training. Regarding the regulation of fuel usage, DBT had a positive impact alongside ET on promoting aerobic glycolysis via significantly decreasing serum lactate by 31.6% and lactic dehydrogenase levels by 61.8%.

**Conclusions:**

This study found that DBT could be considered a promising sports ergogenic aid for athletic population or fitness enthusiasts. Future work focussing on isolating the bioactive components that truly provide the ergogenic effects would be of interest.

## Introduction

In the sports field, scientists have been fascinated with the investigation of the benefits and safety of medicinal herbal products and supplements for enhancing performance or relieving pain. Several herbs have been well studied, and their efficacy has been acknowledged by scientific evidence. For instance, ginseng has a positive effect on stress adaptation and has been found to increase exercise duration until exhaustion (Chen et al. 2012). Ephedra and guarana stimulate the sympathetic nervous system, thereby increasing mental vigilance and fatigue resistance (Sellami et al. 2018).

Recent studies have also highlighted the potential performance-enhancing effect of one herb commonly used in Chinese cuisine, Danggui (DG; Radix *Angelica sinensis* (Oliv.) Diels, [Umbelliferae]) (Yeh et al. 2014; Chang et al. 2016). In traditional Chinese medicine theory, few are used as a single ingredient. Despite the controversy, herbal formulations (multi-ingredients) are generally believed to produce synergic effects and reduce adverse effects (Zhou et al. 2016). One of the most popular DG formulae is Danggui Buxue Tang (DBT). According to the Taiwan Herbal Pharmacopoeia, the traditional formula of DBT is composed of DG and Huangqi [HQ; Radix *Astragalus membranaceus* (Fisch.) Bge. var. *mongholicus* (Bge.) Hsiao (Leguminosae)] at a ratio of 1:5 (Committee on Chinese Medicine and Pharmacy 2016). Nevertheless, Radix *Hedysarum polybotrys* Hand.-Mazz. (Leguminosae) is usually used as a substitute for Radix *Astragali* in the Taiwanese clinic and market due to its similar function and extraordinary taste and flavour (Lu et al. 2009).

There are several major mechanisms involved in the effects of supplements or drugs that may augment physical performance. Boosting anabolism can result in increased muscle mass and muscular strength (Willoughby et al. 2007); stimulating activity of the central nervous system can elevate heart rate and blood pressure and reduce tiredness and fatigue (Avois et al. 2006); regulating fuel metabolism can improve exercise capacity and prolong duration until exhaustion (Ormsbee et al. 2014). Although both animal and human studies have highlighted a dramatic improvement in performance after DBT supplementation (Liu et al. 2011; Chang et al. 2018), its action in terms of biochemical regulation is not yet well understood. According to a recent metabolomics research (Miao et al. 2018), five major metabolic pathways were involved in DBT supplementation on fatigued mice: (1) phenylalanine, tyrosine and tryptophan metabolism, (2) glycine, serine, and threonine metabolism, (3) glyoxylate and dicarboxylate metabolism, (4) pyruvate metabolism, and (5) the Krebs cycle. Consequently, we deduce that DBT more likely alters the energy expenditure during exercise.

Fatigue and impaired muscle function during exercise are commonly linked to muscle glycogen depletion and lactate production (Ortenblad et al. 2013). However, lactate is no longer considered a waste product of anaerobic glycolysis, and in fact, it is an energy source that triggers adaptations in response to high-intensity exercise (Nalbandian and Takeda 2016). Endurance training could benefit the metabolic adaptations by adjusting fuel selection and improving energy expenditure, further resulting in a reduction of lactate (Favier et al. 1986). Rather than depending solely on anaerobic glycolysis during exercise, mitochondrial respiration is the preferred system that functions to replenish ATP. Hence, fuel can be obtained from different sources (fatty acids and carbohydrates) within and outside the muscle (Baker et al. 2010).

This work attempts to understand the effects of DBT supplementation on physical performance and exercise-related biochemical parameters, as well as to monitor its safety in swimming-trained rats. We also investigated whether supplementation with the combined herbal formula DBT was more efficacious than DG alone when each was combined with exercise training.

## Materials and methods

### Preparation of herbal extracts

**Figure 1.**
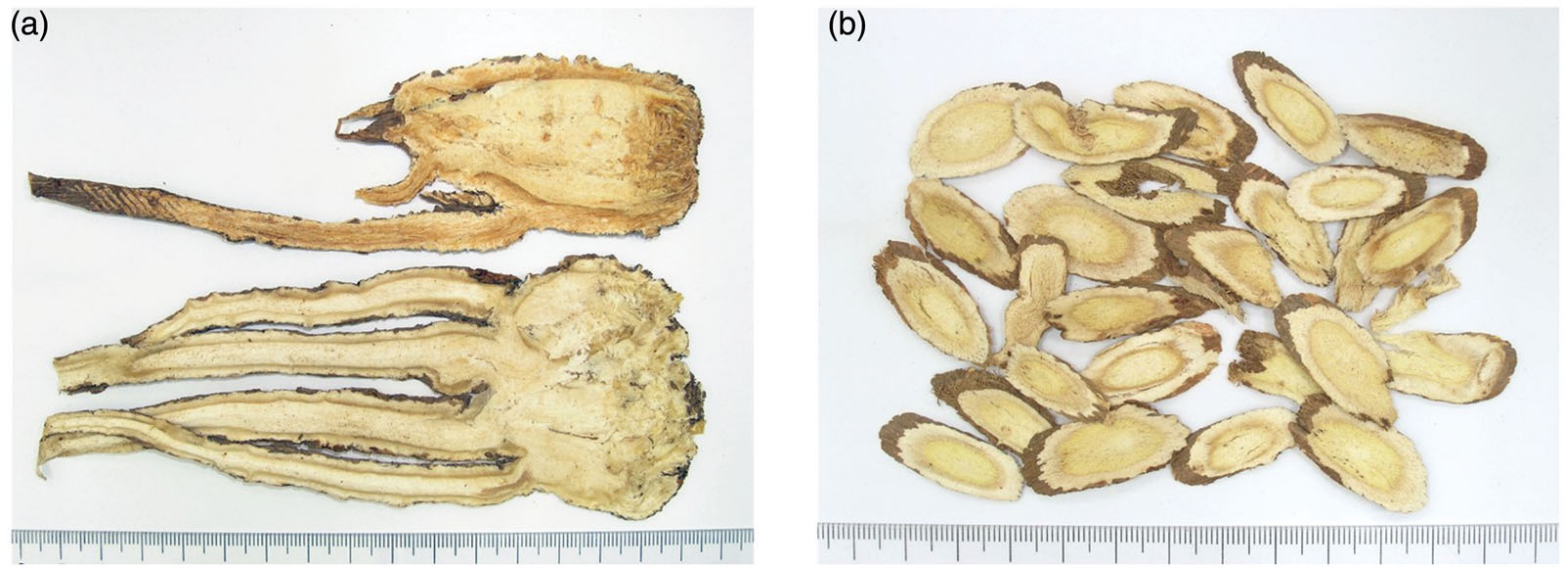
Crude slices of (a) *Angelica sinensis* and (b) *Hedysarum polybotrys*.

Crude slices of herbal material were purchased from a local Chinese medicinal herbs store in Kaohsiung in November 2017. Authentication of the herbs was performed by Mr. Chang-Ming Cheng from Brion Research Institute of Taiwan (New Taipei City, Taiwan) under microscopic observation with the reference criteria (Chinese Pharmacopoeia Commission 2015; Committee on Chinese Medicine and Pharmacy 2016). The herbs (Figure 1) were authenticated as the dried root of *Angelica sinensis* and *Hedysarum polybotrys*, respectively. This particular batch of herbal materials used in this study has not be deposited in a publicly available herbarium.

To prepare the herbal extract, the crude materials (600 g Radix *Angelica sinensis* for the DG extract; 600 g Radix *Angelica sinensis* and 3000 g Radix *Hedysarum* for the DBT extract) were first extracted with 95% ethanol for 15 h at a solid-to-liquid ratio of 1:4. The first portion of ethanolic extract was removed and collected. The residue material then underwent the second extraction with boiling water for 3 h at the same solid-to-liquid ratio, and the second portion of the extract was collected. The two portions were combined and concentrated through rotary evaporation at 45 °C. The yield of the final extract was approximately 30%.

### Phytochemical analysis of herbal extracts

Total polysaccharides, ferulic acid, ligustilide, and *n*-butylidenephthalide content in the DG and DBT extracts were quantitatively analysed using spectrophotometric and chromatographic methods as previously described (Chang et al. 2018).

### Animals

The animal study protocol was reviewed and approved by the Institutional Animal Care and Use Committee of Kaohsiung Medical University (Kaohsiung, Taiwan; approval number: 105233). Five-week-old male Wistar rats were purchased from Biolasco (Taipei, Taiwan) and kept in regularly cleaned plastic cages, with a maximum of 4 animals per cage. Food (LabDiet 5001 rodent diet, St. Louis, MO, USA) and pure water were provided *ad libitum*.

### Study design

**Figure 2.**
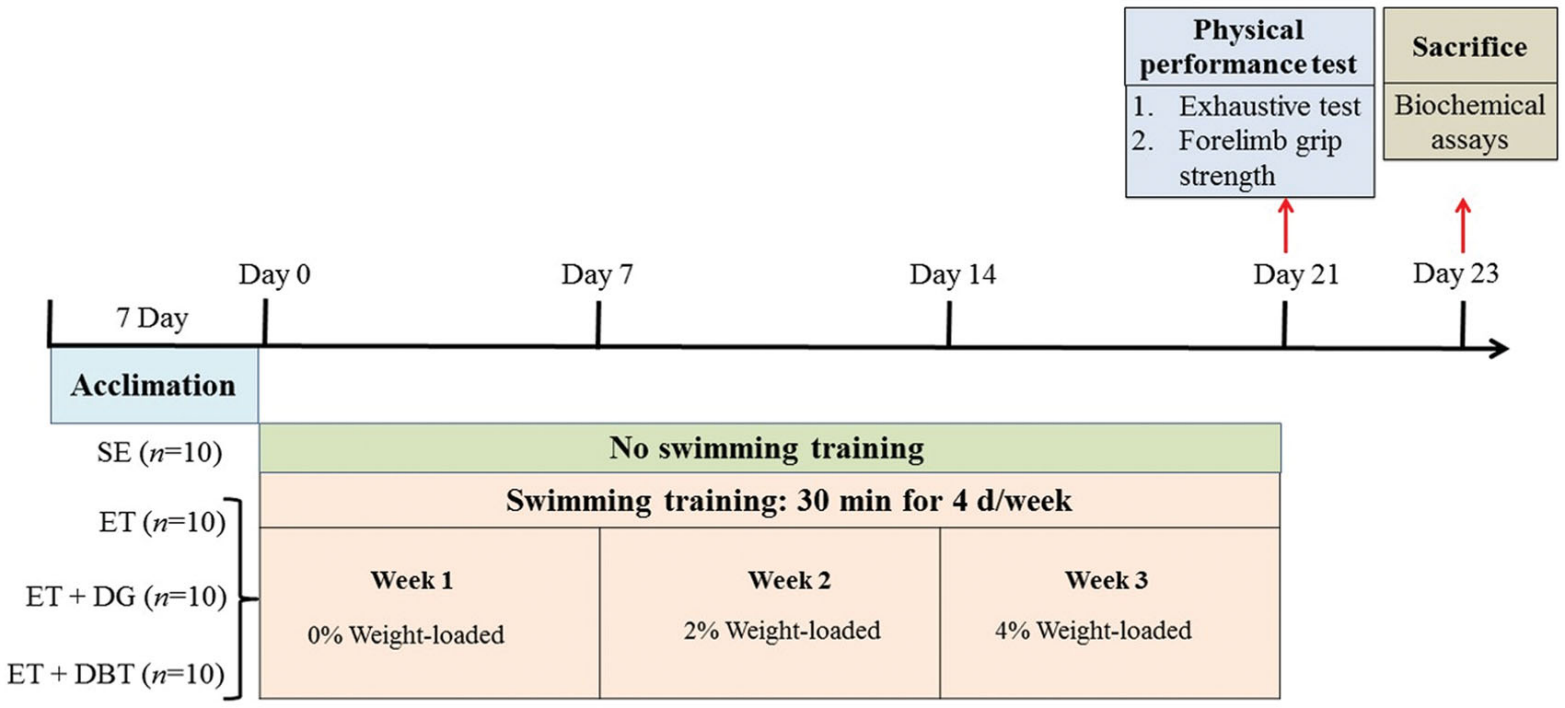
Experimental design of the study.

After a 1-week acclimation period, 40 animals were randomly assigned to four groups: sedentary (SE; *n* = 10), exercise training (ET; *n* = 10), ET supplemented with 0.3 g/kg rat/d DG extract (ET + DG; *n* = 10), and ET supplemented with 1.8 g/kg rat/d DBT extract (ET + DBT; *n* = 10). The herbal extract solutions were prepared with pure water at concentrations of 0.03 and 0.18 g/mL for DG and DBT, respectively. A volume based on their individual body weight (10 mL/kg rat/d) of the herbal solution or pure water (for SE and ET) was fed daily through oral gavage for a period of 21 days. Body weight was recorded semi-weekly, and food intake was recorded weekly. The experimental design is shown in Figure 2.

The exercised groups were subjected to a protocol of swimming training in a transparent water container measuring 30 cm in depth measuring at 24 ± 2 °C. They performed 30 min of swimming 4 d/wk over the same period of supplementation (21 days) with an additional load that increased each week (0, 2, 4% body weight). The loading was carried out via fastening a soft strap with metal nuts around the chest of rats. After each session, animals were towel-dried and returned to their cages.

### Physical performance tests

The physical performance tests, consisting of the forelimb grip strength test and exhaustive swimming test as previously described (Chang et al. 2016), were performed 72 h before sacrifice. Briefly, the maximal forelimb grip strength for each animal was first tested in triplicate using a grip strength metre (BIO-GS3, Bioseb, France). Then, the mean value was calculated and recorded. In the exhaustive swimming test, the rats were loaded with 10% of their body weight and the other parameters were set as the same as swimming training. The rats in SE were allowed to familiarise and reduce stress against water by swimming a short period of time without any loading days prior to the exhaustive swimming test. The swimming duration until exhaustion was recorded.

### Blood and tissue sampling

At the end of the study, animals were sacrificed between 9 and 12 noon under fed condition. Rats were anaesthetized with 5% isoflurane and euthanized by exsanguination. Blood specimens were collected from the abdominal aortas and separated into EDTA tubes and serum-separating tubes to obtain whole blood and serum, respectively. Tissue, including liver, kidney, lung, heart, and calf muscle samples, was also collected and fixed for histological evaluation in 10% neutral buffered formalin.

### Biochemical assays

Biochemical analyses were performed in accordance with the manufacturers’ instructions. The levels of complete blood count were assayed on a Sysmex XN-1000 Haematology Analyser (Kobe, Hyogo, Japan). Serum aspartate aminotransferase (AST), alanine aminotransferase (ALT), blood urea nitrogen (BUN), creatinine, uric acid, lactic dehydrogenase (LDH), ammonia, and creatine phosphokinase (CPK) levels were assayed on a Beckman Coulter UniCel DxC 800 (Brea, CA, USA). Serum lactate levels were measured using Roche Cobas c 501 (Mannheim, Germany). Muscle glycogen contents were measured using the Glycogen Colorimetric Assay Kit II (BioVision, Milpitas, CA, USA).

### Histological staining

The staining methods were performed as previously described (Chang et al. 2016). Sections of muscle tissue were stained with periodic acid solution and Schiff’s reagent (PAS) to detect the presence of glycogen. Sections of heart, lung, liver, kidney, and muscle tissues were stained with haematoxylin and eosin to observe the histology.

### Statistical analysis

SPSS 22.0 (International Business Machines Corporation, Armonk, NY, USA) was used for statistical analyses. Data are expressed as the mean ± standard error of the mean (SEM). One-way ANOVA was performed with Tukey’s posttest to compare the parameters between groups. A *p* value < 0.05 was considered statistically significant.

## Results

### Phytochemical analysis of herbal extracts

The bioactive components were identified and confirmed as total polysaccharides (107.8 and 184.1 mg/g for DG and DBT, respectively), ferulic acid (0.14 and 0.03 mg/g for DG and DBT), ligustilide (0.83 and 0.22 mg/g for DG and DBT), and *n*-butylidenephthalide (3.85 and 0.52 mg/g for DG and DBT). Therefore, the daily administration of total polysaccharides was 32.3 mg/kg for the ET + DG group and 331.4 mg/kg for the ET + DBT group.

### Herbal extract supplementation on physical performance

**Figure 3.**
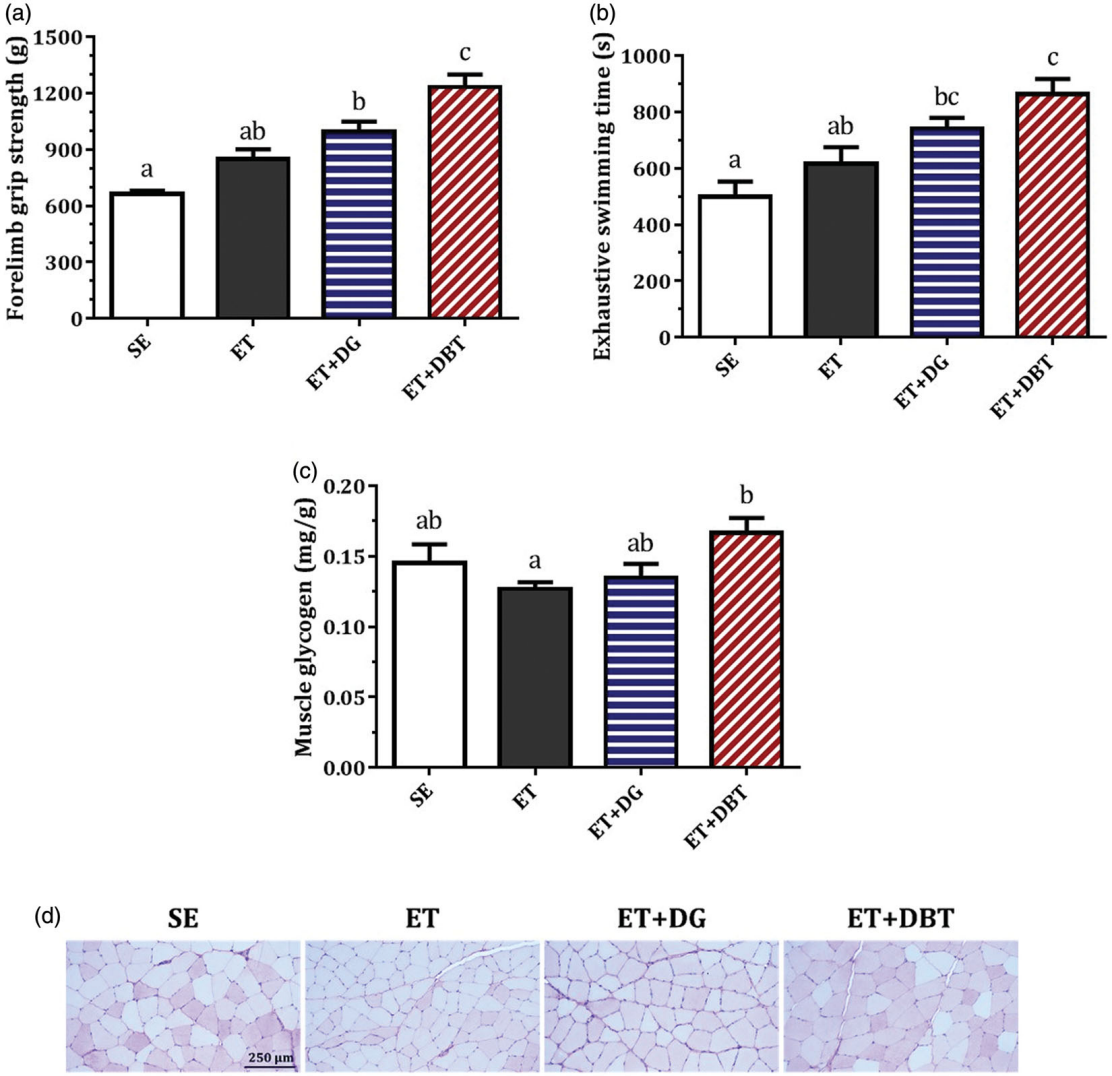
Effects of exercise training and herbal extract supplementation on (a) forelimb grip strength, (b) exhaustive swimming time, (c) muscle glycogen content, and (d) histology of muscle tissues (PAS staining of glycogen, presented in magenta). Values are presented as mean ± SEM for *n* = 10 rats. Columns with different letters (a, b, c) are significantly different at *p* < 0.05.

Two similar trends (Figure 3(a,b)) in terms of performance improvement were observed in both the forelimb grip strength results (representing muscular strength) and exhaustive swimming time results (representing endurance capacity). As comparing ET with SE, the 21-day training programme was not able to bring a striking improvement in muscular strength or endurance capacity. However, we observed a slight increase in grip strength (27.9%) and a prolongation in swimming time (23.2%) in the non-supplemented exercise animals.

DG supplementation remarkably increased the grip strength by 49.7% (*p* < 0.001) and the swimming time by 48.4% (*p* < 0.05) compared with SE. Furthermore, DBT supplementation produced dramatic improvements in grip strength by 85.7% (*p* < 0.001) and swimming time by 72.7% (*p* < 0.001) compared with SE. In particular, the grip strength in DBT-treated rats was significantly better than the rats treated with DG alone (*p* < 0.05).

### Herbal extract supplementation on muscle glycogen contents

Glycogen storage in muscle was quantitatively analysed and further confirmed by PAS staining; glycogen is presented in magenta (Figure 3(c,d)). In the ET group, the glycogen contents were 12.4% lower than in the SE group (without training). While DG supplementation did not affect glycogen levels much, DBT significantly improved glycogen storage (*p* < 0.05) and preserved a certain amount of glycogen in the muscle cells compared with the ET group.

### Herbal extract supplementation on lactate and lactic dehydrogenase levels

**Figure 4.**
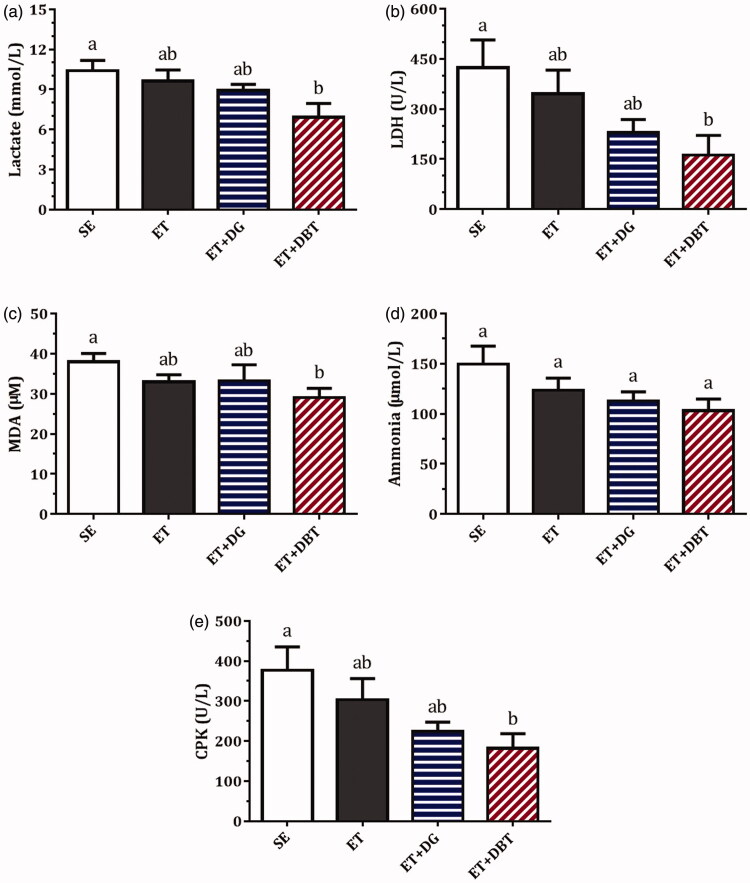
Effects of exercise training and herbal extract supplementation on serum (a) lactate (b) LDH, (c) MDA, (d) ammonia, and (e) CPK. Values are presented as mean ± SEM for *n* = 10 rats. Columns with different letters (a, b, c) are significantly different at *p* < 0.05.

As shown in Figure 4, the lactate levels were slightly reduced by 9.9% and 14.2% in the ET and ET + DG groups (*p* > 0.05) compared with the SE group; nonetheless, a significant reduction of 31.6% was observed in the ET + DBT group (*p* < 0.05). LDH activities were found to be suppressed after ET (by 18.3%) and ET + DG (by 45.8%) treatment, but the results were not significant (*p* > 0.05). Notably, ET + DBT treatment led to a substantial reduction of 61.8% (*p* < 0.05).

### Herbal extract supplementation on other exercise-related parameters

Levels of malondialdehyde (MDA), ammonia, and creatine phosphokinase, which are parameters commonly used for examining exercise-induced damage or fatigue, are shown in Figure 4. MDA levels, a marker for oxidative stress, did not differ between the SE, ET, and ET + DG groups. However, lower amounts of MDA were found in the ET + DBT group (*p* < 0.01) compared with the SE group. Although decreases in ammonia concentrations were observed in the ET group (by 17.4%), ET + DG group (by 24.7%), and ET + DBT group (by 31.0%), relative to the SE group, no significant difference appeared between each of them. CPK levels were also decreased in the ET group (19.4%), ET + DG group (40.6%), and ET + DBT group (48.1%), compared with the SE group; however, only ET + DBT treatment resulted in a significant effect (*p* < 0.05).

### Short-term safety evaluation of herbal extract supplementation

**Figure 5.**
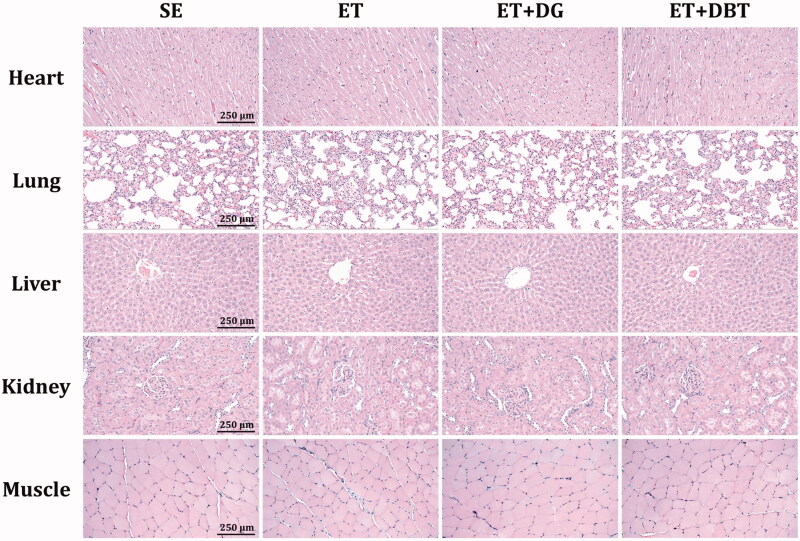
Effects of exercise training and herbal extract supplementation on the histology of heart, lung, liver, kidney, and muscle tissue (H&E staining).

No differences were found in the body weights and food intake throughout the study period, as well as the heart, lung, liver, kidney, muscle tissue weights (Table 1). The complete blood count analysis (Table 2) and markers for liver function (i.e., aspartate aminotransferase and alanine aminotransferase) and kidney function (i.e., blood urea nitrogen, creatinine, and uric acid) (Table 3) did not differ between groups. In the histological staining shown in Figure 5, we did not observe any hypertrophy or hyperplasia in heart cardiomyocytes. Lung tissue displayed normal bronchiole and alveoli. Liver tissue exhibited the normal structure of the hepatic cords and vascular sinusoids. Kidney tissue showed the normal arrangement of renal glomerulus and tubules. The appearance of the rhabdomyocytes in muscle tissue did not differ among treatments. Taken together, the results indicated that short-term swimming training, DG extract, and DBT extract would not cause any foreseeable toxicity.

**Table 1.** Effects of exercise training and herbal extract supplementation on body weight, food intake, and tissue weight.

	SE	ET	ET + DG	ET + DBT
Initial body weight (g)	222.3 ± 3.5	223.5 ± 2.3	223 ± 2.6	221.6 ± 2.4
Day-3 body weight (g)	241.0 ± 4.3	242.3 ± 2.2	239.6 ± 3.5	241.1 ± 3.3
Day-7 body weight (g)	279.2 ± 5.9	275.5 ± 2.5	276.2 ± 4.1	275.8 ± 3.5
Day-10 body weight (g)	289.6 ± 6.4	286.5 ± 3.2	283.6 ± 4.5	286.7 ± 3.5
Day-14 body weight (g)	327.2 ± 8.0	315.0 ± 5.1	317.4 ± 5.5	319.8 ± 3.8
Day-17 body weight (g)	340.7 ± 9.5	338.9 ± 3.2	341.0 ± 7.1	338.1 ± 3.3
Final body weight (g)	358.6 ± 10.0	358.2 ± 4.5	357.1 ± 7.3	356.1 ± 4.1
Week-1 food intake (g/d/rat)	24.33 ± 0.54	24.71 ± 0.16	24.98 ± 0.06	24.76 ± 0.54
Week-2 food intake (g/d/rat)	26.39 ± 0.67	25.14 ± 0.06	25.93 ± 0.06	25.08 ± 0.39
Week-3 food intake (g/d/rat)	26.17 ± 0.85	26.86 ± 0.43	27.39 ± 0.15	26.47 ± 0.69
Liver (g)	10.45 ± 0.32	10.45 ± 0.29	10.25 ± 0.34	10.47 ± 0.25
Kidney (g)	2.47 ± 0.05	2.54 ± 0.08	2.46 ± 0.05	2.56 ± 0.06
Lung (g)	1.43 ± 0.05	1.46 ± 0.03	1.57 ± 0.06	1.42 ± 0.02
Heart (g)	1.36 ± 0.06	1.36 ± 0.05	1.43 ± 0.04	1.43 ± 0.04
Muscle (g)	4.71 ± 0.17	4.82 ± 0.10	4.70 ± 0.09	4.54 ± 0.08

Values are presented as mean ± SEM for *n* = 10 rats. All data were not significant different between groups analysed by one-way ANOVA with Tukey’s posttest (*p* > 0.05).

**Table 2.** Effects of exercise training and herbal extract supplementation on complete blood count analysis.

	SE	ET	ET + DG	ET + DBT
WBC (cells/mm^3^)	3706 ± 694	3774 ± 415	3695 ± 330	4627 ± 514
Neutrophils (%)	14.9 ± 1.1	17.4 ± 0.9	17.4 ± 1.7	14.7 ± 1.3
Lymphocytes (%)	80.5 ± 1.1	77.7 ± 0.89	77.2 ± 2.1	80.3 ± 5.0
Monocytes (%)	2.35 ± 0.37	2.32 ± 0.42	2.76 ± 0.59	2.78 ± 0.58
Eosinophils (%)	2.23 ± 0.24	2.57 ± 0.25	2.40 ± 0.22	2.12 ± 0.21
Basophils (%)	0.04 ± 0.04	0.11 ± 0.0	0.24 ± 0.08	0.15 ± 0.05
RBC (×10^6^/mm^3^)	6.70 ± 0.65	7.80 ± 0.33	8.10 ± 1.11	8.29 ± 0.52
Haemoglobin (g/mL)	13.1 ± 1.2	15.4 ± 0.8	15.8 ± 2.2	16.4 ± 1.0
Haematocrit (%)	41.1 ± 4.0	48.4 ± 2.2	49.0 ± 6.7	50.9 ± 3.3
MCV (fL)	61.2 ± 0.6	62.0 ± 0.6	60.5 ± 0.4	61.4 ± 0.5
MCH (pg)	19.6 ± 0.2	19.7 ± 0.2	19.5 ± 0.1	19.8 ± 0.2
MCHC (%)	32.1 ± 0.3	31.8 ± 0.2	32.3 ± 0.1	32.3 ± 0.2
Platelet (×10^3^/mm^3^)	838.1 ± 113.0	834.2 ± 70.3	895.5 ± 126.7	943.0 ± 60.6

Values are presented as mean ± SEM for *n* = 10 rats. All data were not significant different between groups analysed by one-way ANOVA with Tukey’s posttest (*p* > 0.05).

**Table 3.** Effects of exercise training and herbal extract supplementation on serum markers of liver function and kidney function.

	SE	ET	ET + DG	ET + DBT
AST (U/L)	78.5 ± 3.1	78.7 ± 4.0	79.7 ± 2.2	73.6 ± 3.3
ALT (U/L)	30.9 ± 1.3	29.5 ± 1.7	29.0 ± 1.3	29.0 ± 1.4
BUN (mg/dL)	13.6 ± 0.4	15.1 ± 1.2	14.0 ± 0.3	13.1 ± 0.4
Creatinine (mg/dL)	0.32 ± 0.02	0.33 ± 0.02	0.31 ± 0.02	0.28 ± 0.01
Uric acid (mg/dL)	1.48 ± 0.20	1.15 ± 0.09	1.16 ± 0.08	0.98 ± 0.13

Values are presented as mean ± SEM for *n* = 10 rats. All data were not significant different between groups analysed by one-way ANOVA with Tukey’s posttest (*p* > 0.05).

## Discussion

### The doses and phytochemical components of DG and DBT

Out of more than 80 traditional Chinese formulae containing DG, DBT is one of the simplest formulae that is composed of DG and HQ in the ratio of 1:5. The majority in terms of the composition of DBT is taken by HQ rather than DG. In the theory of traditional Chinese Medicine (TCM), an herbal decoction may consist up to four elements, namely Jun (king or master), Chen (minister), Zuo (assistant), and Shi (servant), which work harmoniously together in order to achieve the optimum therapeutic purposes. In DBT, HQ can nourish one’s ‘Qi’ while DG can replenish one’s ‘Blood’. HQ is considered as a Jun whereas DG acts as a Chen (Dong et al. 2006). According to (Zhang et al. 2012) ‘Qi’ and ‘Blood’ are inextricable in the TCM theory. The ‘Blood’ constitutes the whole liquid part of the body energy (body fluids). The ‘Qi’ is imaginary energy and can be refined as nutritious substances that support vital life activities. The ‘Blood’ cannot be self-generated and which requires the stimulation by the ‘Qi’.

A previous study (Song et al. 2004) unveiled an interesting synergic effect of DG and HQ under decoction. The constituents ferulic acid and ligustilide from DG can be oxidised and degraded due to heat. However, the combination of DG and HQ ameliorated their oxidisation process during boiling thereby produced a higher yield compared to DG alone. Having a cocktail of various chemicals in the DBT decoction not only improved the stability but also the extraction recovery of the active constituents. Based on the above, it might explain the reason for the ancient belief, at least in part, that herbs tend to be given in a formulation to obtain the maximal pharmacological activity.

According to the Taiwan Herbal Pharmacopoeia (Committee on Chinese Medicine and Pharmacy 2016), the recommendation of daily intake for crude DG is 5–15 g. Considering the yield of the extract is 30% of crude DG, the dose of DG extract should be within 25-75 mg/kg human/d for a 60 kg human being. In the current study, we chose the oral dose of 300 mg/kg rat/d that has been shown to be pharmacological effective in rats (Circosta et al. 2006; Zhang et al. 2010; Chang et al. 2016). Under the dose conversion between rat and human (Nair and Jacob 2016), 300 mg/kg rat/d is equivalent to 48.4 mg/kg human/d which the dose is within the recommended range for traditional use. DBT consists one portion of DG and five portions of HQ. As a consequence, 1800 mg/kg rat/d of DBT extract was given to rats.

Ferulic acid, ligustilide, and *n*-butylidenephthalide are currently listed as quality control markers for DG (Chinese Pharmacopoeia Commission 2015; Committee on Chinese Medicine and Pharmacy 2016). One study found that administering ferulic acid was able to stimulate endurance exercise capacity and increase the activity of hepatic antioxidant enzymes (e.g., superoxide dismutase, catalase, and glutathione-*S*-transferase) in mice (You et al. 2009). Notwithstanding this, recent studies further showed that the water-soluble portion, polysaccharides, has been regarded as the crucial modifiers for biological response (Sun et al. 2005; Zhao et al. 2016). Polysaccharides from many herbs, for instance, *Millettiae speciosae* Champ. (Leguminosae) (Zhao et al. 2015), *Dendrobium officinale* Kimura et Migo (Orchidaceae) (Wei et al. 2017), okra (*Abelmoschus esculentus* (L.) Moench, Malvaceae) (Xia et al. 2015), and maca (*Lepidium meyenii* Walp., Brassicaceae) (Li et al. 2018), have been revealed as the excellent choices to tackle fatigue in rodent swimming models. PhytoHealth PG2®, the injectable polysaccharides isolated from HQ, is launched by 2012 and approved for treating fatigue in the patients with cancer. The Phase IV study has demonstrated that HQ polysaccharides is particularly effective towards the alleviation of fatigue in more than 60% treated cancer patients (Wang et al. 2019).

In our study, we observed that the content per unit of total polysaccharides in DBT (184.1 mg/g) was higher than that in DG (107.8 mg/g). This result may indicate that richer polysaccharides present in HQ than in DG, and further relate to the superior beneficial effects observed in DBT-treated rats.

### DBT acting on physical performance, muscle glycogen and glycolysis

Our results showed that the 21-day training programme (without supplementation) was not able to bring a striking improvement in muscular strength or endurance capacity. Such findings were in accordance with some previous studies. Wei et al. (2019) implemented a 42-day swimming programme with additional loading of 3–35% bodyweight, the performance in forelimb grip strength and exhaustive time did not differ between the sedentary and the exercised mice. Yuan et al. (2019) revealed a slight prolong in mice exhaustive swimming time after a swimming training of 20 min/d for 28 days; unfortunately, the improvement was insignificant. Conversely, a study (Venditti and Di Meo 1996) revealed that the endurance time was significantly longer in the trained rats (450 ± 35 min) than in the untrained rats (294 ± 32 min). The effective training comprised a 10-week swimming programme for up to 90 min for 5 d/week. Based on above, we might come to a conclusion of insufficient length and intensity of our swimming programme that is unable to alter the exercise capacity.

However, the herbal extract supplementation has led to an enhancement of the exercise capacity. The effects of DG, HQ, and DBT acting on physical performance have been published elsewhere. Yeh et al. (2014) gave DG powdered product to mice for six weeks, the weight-loaded swimming time was increased up by 81%. Similar results could also be seen in the study administering DG ethanol extract (Chang et al. 2016). The rat swimming time was increased by 95.6% after four-week treatment. Hu and Hou (2003) found that a 7-day HQ water extract administration prolonged the running time by 31% in mice cardiac hypertrophy model. Kuo et al. (2009) evidenced that a 6-week treatment of HQ flavonoids could also reverse the chronic fatigue syndrome via improving endurance capacity in rats. Hence, it is not surprising that DBT decoction, combining DG and HQ, has remarkable therapeutic effects on antifatigue (Liu et al. 2011; Miao et al. 2018). The investigation of the underlying mechanism is currently of great interest in both ethnopharmacology and sports nutrition field.

High carbohydrate availability is usually recommended to prolong exercise activities. To generate energy for exercise, glucose and glycogen are converted to pyruvate. One fate of pyruvate is converted to lactate, which results in two molecules of ATP per glucose molecule (anaerobic glycolysis). Lactic dehydrogenase (LDH) acts as the enzyme catalysing the conversion between lactate to pyruvate and back. Anaerobic glycolysis can barely sustain continued muscle activity for a long time. However, with an increase of oxygen uptake, glucose molecules can generate many times more ATP via the Krebs cycle within the mitochondria (aerobic glycolysis). Aerobic metabolism is thus a preferred process for supplying the primary fuel required by contracting muscles (Mul et al. 2015).

One study revealed that HQ polysaccharide was able to improve the energy metabolism in the rats treated with a high-fat and low-protein diet plus exhaustive swimming programme (Wang et al. 2018). Citric acid, an important intermediate of the Krebs cycle, reflects the condition of energy supply from glycometabolism, and lipid and amino acid metabolism. After HQ polysaccharide treatment, serum citric acid was gradually restored and the lactic acid level was reduced compared to the model group.

Another study investigated the effects of DBT in fatigued mice by means of a metabolomics approach (Miao et al. 2018). Pyruvate and lactate involving in the glycolytic pathway were downregulated and citric acid involving in the Krebs cycle was upregulated after the DBT treatment. This suggested that DBT could enhance mitochondrial function and ATP generation by affecting the node metabolites and further changing the metabolism pathway networks.

In our study, we administered DBT extract (combined water and ethanolic extract) to the swimming training rats. Twenty-one-day DBT treatment resulted in a substantial augmentation in terms of physical performance. By viewing the preservation of muscle glycogen and the reduction of serum lactate and LDH levels, we confirmed that DBT increased the physiological adaptations in response to exercise by altering energy expenditure. DBT supplementation allowed the body to cover the energy demand during exercise as well as improved its capacity for sustaining a high-rate of aerobic glycolysis.

## Conclusions

This study found that DBT, one of the most popular Chinese herbal formulae, is a promising sports ergogenic aid. Under a regular swimming training programme, DBT remarkably increased forelimb grip strength and prolonged exhaustive swimming time in rats. The abundant content of polysaccharides in DBT might contribute to the promotion of sparing muscle glycogen. DBT-triggered metabolic adaptations to energy expenditure for sustaining aerobic glycolysis during exercise via reducing LDH and lactate levels. Moreover, alongside its potential antioxidant and antifatigue properties, short-term DBT supplementation resulted in a remarkable increase in exercise capacity without foreseeable safety concerns. Overall, DBT resulted in a better ergogenic effect than using DG alone.

## References

[CIT0001] Avois L, Robinson N, Saudan C, Baume N, Mangin P, Saugy M. 2006. Central nervous system stimulants and sport practice. Br J Sports Med. 40(Supplement 1):i16–i20. 16799095 10.1136/bjsm.2006.027557 PMC2657493

[CIT0002] Baker JS, McCormick MC, Robergs RA. 2010. Interaction among skeletal muscle metabolic energy systems during intense exercise. J Nutr Metab. 2010:905612. 21188163 10.1155/2010/905612 PMC3005844

[CIT0003] Chang CW, Chen YM, Hsu YJ, Huang CC, Wu YT, Hsu MC. 2016. Protective effects of the roots of *Angelica sinensis* on strenuous exercise-induced sports anemia in rats. J Ethnopharmacol. 193:169–178. 27497636 10.1016/j.jep.2016.08.010

[CIT0004] Chang CW, Chen CY, Yen CC, Wu YT, Hsu MC. 2018. Repressed exercise-induced hepcidin levels after Danggui Buxue Tang supplementation in male recreational runners. Nutrients. 10(9):1318. 10.3390/nu10091318 PMC6165347 30231484

[CIT0005] Chen CK, Muhamad AS, Ooi FK. 2012. Herbs in exercise and sports. J Physiol Anthropol. 31:4 22738233 10.1186/1880-6805-31-4 PMC3375032

[CIT0006] Chinese Pharmacopoeia Commission. 2015. Pharmacopoeia of the People’s Republic of China. China: Chinese Pharmacopoeia Commission. http://www.chp.org.cn/.

[CIT0007] Circosta C, Pasquale RD, Palumbo DR, Samperi S, Occhiuto F. 2006. Estrogenic activity of standardized extract of *Angelica sinensis*. Phytother Res. 20(8):665–669. 16691630 10.1002/ptr.1928

[CIT0008] Committee on Chinese Medicine and Pharmacy. 2016. Taiwan herbal pharmacopeia. Taiwan: Ministry of Health and Welfare. https://dep.mohw.gov.tw/.

[CIT0009] Dong TT, Zhao KJ, Gao QT, Ji ZN, Zhu TT, Li J, Duan R, Cheung AW, Tsim KW. 2006. Chemical and biological assessment of a Chinese herbal decoction containing Radix Astragali and Radix Angelicae sinensis: determination of drug ratio in having optimized properties. J Agric Food Chem. 54(7):2767–2774. 16569074 10.1021/jf053163l

[CIT0010] Favier RJ, Constable SH, Chen M, Holloszy JO. 1986. Endurance exercise training reduces lactate production. J Appl Physiol. 61(3):885–889. 3759772 10.1152/jappl.1986.61.3.885

[CIT0011] Hu YC, Hou JY. 2003. Effect of zhimu and huangqi on cardiac hypertrophy and response to stimulation in mice. Zhongguo Zhong Yao Za Zhi. 28(4):369–374. 15139154

[CIT0012] Kuo YH, Tsai WJ, Loke SH, Wu TS, Chiou WF. 2009. *Astragalus membranaceus* flavonoids (AMF) ameliorate chronic fatigue syndrome induced by food intake restriction plus forced swimming. J Ethnopharmacol. 122(1):28–34. 19103273 10.1016/j.jep.2008.11.025

[CIT0013] Li Y, Xin Y, Xu F, Zheng M, Xi X, Cui X, Cao H, Guo H, Han C. 2018. Maca polysaccharides: extraction optimization, structural features and anti-fatigue activities. Int J Biol Macromol. 115:618–624. 29665394 10.1016/j.ijbiomac.2018.04.063

[CIT0014] Liu Y, Zhang HG, Li XH. 2011. A Chinese herbal decoction, Danggui Buxue Tang, improves chronic fatigue syndrome induced by food restriction and forced swimming in rats. Phytother Res. 25(12):1825–1832. 21495102 10.1002/ptr.3499

[CIT0015] Lu K-T, Lee H-C, Liu F-S, Lo C-F, Lin J-H. 2009. Discriminating Astragali Radix from Hedysarum Radix in Chinese medicine preparations using nested PCR and DNA sequencing methods. J Food Drug Anal. 17:380–385.

[CIT0016] Miao X, Xiao B, Shui S, Yang J, Huang R, Dong J. 2018. Metabolomics analysis of serum reveals the effect of Danggui Buxue Tang on fatigued mice induced by exhausting physical exercise. J Pharm Biomed Anal. 151:301–309. 29413978 10.1016/j.jpba.2018.01.028

[CIT0017] Mul JD, Stanford KI, Hirshman MF, Goodyear LJ. 2015. Exercise and regulation of carbohydrate metabolism. Prog Mol Biol Transl Sci. 135:17–37. 26477909 10.1016/bs.pmbts.2015.07.020 PMC4727532

[CIT0018] Nair AB, Jacob S. 2016. A simple practice guide for dose conversion between animals and human. J Basic Clin Pharm. 7(2):27–31. 27057123 10.4103/0976-0105.177703 PMC4804402

[CIT0019] Nalbandian M, Takeda M. 2016. Lactate as a signaling molecule that regulates exercise-induced adaptations. Biology. 5(4):38. 10.3390/biology5040038 PMC5192418 27740597

[CIT0020] Ormsbee MJ, Bach CW, Baur DA. 2014. Pre-exercise nutrition: the role of macronutrients, modified starches and supplements on metabolism and endurance performance. Nutrients. 6(5):1782–1808. 24787031 10.3390/nu6051782 PMC4042570

[CIT0021] Ortenblad N, Westerblad H, Nielsen J. 2013. Muscle glycogen stores and fatigue. J Physiol (Lond). 591(18):4405–4413. 23652590 10.1113/jphysiol.2013.251629 PMC3784189

[CIT0022] Sellami M, Slimeni O, Pokrywka A, Kuvacic G, L DH, Milic M, Padulo J. 2018. Herbal medicine for sports: a review. J Int Soc Sports Nutr. 15:14 29568244 10.1186/s12970-018-0218-y PMC5856322

[CIT0023] Song ZH, Ji ZN, Lo CK, Dong TT, Zhao KJ, Olive T, Haines CJ, Kung SD, Tsim KW. 2004. Chemical and biological assessment of a traditional Chinese herbal decoction prepared from Radix Astragali and Radix Angelicae Sinensis: orthogonal array design to optimize the extraction of chemical constituents. Planta Med. 70(12):1222–1227. 15643561 10.1055/s-2004-835855

[CIT0024] Sun Y, Tang J, Gu X, Li D. 2005. Water-soluble polysaccharides from *Angelica sinensis* (Oliv.) Diels: preparation, characterization and bioactivity. Int J Biol Macromol. 36(5):283–289. 16129482 10.1016/j.ijbiomac.2005.07.005

[CIT0025] Venditti P, Di Meo S. 1996. Antioxidants, tissue damage, and endurance in trained and untrained young male rats. Arch Biochem Biophys. 331(1):63–68. 8660684 10.1006/abbi.1996.0283

[CIT0026] Wang CH, Lin CY, Chen JS, Ho CL, Rau KM, Tsai JT, Chang CS, Yeh SP, Cheng CF, Lai YL. 2019. Karnofsky performance status as a predictive factor for cancer-related fatigue treatment with *Astragalus* polysaccharides (PG2) injection-a double blind, multi-center, randomized phase IV study. Cancers (Basel). 11(2):128. 10.3390/cancers11020128 PMC6406819 30678249

[CIT0027] Wang H, Liu A, Zhao W, Zhao H, Gong L, Chen E, Cui N, Ji X, Wang S, Jiang H. 2018. Metabolomics research reveals the mechanism of action of *Astragalus* polysaccharide in rats with digestive system disorders. Molecules. 23(12):3333. 10.3390/molecules23123333 PMC6321338 30558291

[CIT0028] Wei W, Li ZP, Zhu T, Fung HY, Wong TL, Wen X, Ma DL, Leung CH, Han QB. 2017. Anti-fatigue effects of the unique polysaccharide marker of *Dendrobium officinale* on BALB/c mice. Molecules. 22(1):155. 10.3390/molecules22010155 PMC6155575 28106808

[CIT0029] Wei L, Wen YT, Lee MC, Ho HM, Huang CC, Hsu YJ. 2019. Effects of isolated soy protein and strength exercise training on exercise performance and biochemical profile in postpartum mice. Metab Clin Exp. 94:18–27. 30731100 10.1016/j.metabol.2019.01.012

[CIT0030] Willoughby DS, Stout JR, Wilborn CD. 2007. Effects of resistance training and protein plus amino acid supplementation on muscle anabolism, mass, and strength. Amino Acids. 32(4):467–477. 16988909 10.1007/s00726-006-0398-7

[CIT0031] Xia F, Zhong Y, Li M, Chang Q, Liao Y, Liu X, Pan R. 2015. Antioxidant and anti-fatigue constituents of Okra. Nutrients. 7(10):8846–8858. 26516905 10.3390/nu7105435 PMC4632455

[CIT0032] Yeh TS, Huang CC, Chuang HL, Hsu MC. 2014. *Angelica sinensis* improves exercise performance and protects against physical fatigue in trained mice. Molecules. 19(4):3926–3939. 24691065 10.3390/molecules19043926 PMC6271504

[CIT0033] You Y, Park J, Yoon HG, Lee YH, Hwang K, Lee J, Kim K, Lee KW, Shim S, Jun W. 2009. Stimulatory effects of ferulic acid on endurance exercise capacity in mice. Biosci Biotechnol Biochem. 73(6):1392–1397. 19502723 10.1271/bbb.90062

[CIT0034] Yuan T, Wu D, Sun K, Tan X, Wang J, Zhao T, Ren B, Zhao B, Liu Z, Liu X. 2019. Anti-fatigue activity of aqueous extracts of *Sonchus arvensis* L. in exercise trained mice. Molecules. 24(6):1168. 10.3390/molecules24061168 PMC6470720 30934545

[CIT0035] Zhang S, He B, Ge J, Li H, Luo X, Zhang H, Li Y, Zhai C, Liu P, Liu X, et al. 2010. Extraction, chemical analysis of *Angelica sinensis* polysaccharides and antioxidant activity of the polysaccharides in ischemia-reperfusion rats. Int J Biol Macromol. 47(4):546–550. 20691723 10.1016/j.ijbiomac.2010.07.012

[CIT0036] Zhang WL, Zheng KY, Zhu KY, Zhan JY, Bi CW, Chen JP, Du CY, Zhao KJ, Lau DT, Dong TT, et al. 2012. Chemical and biological assessment of *Angelica* herbal decoction: comparison of different preparations during historical applications. Phytomedicine. 19(11):1042–1048. 22902230 10.1016/j.phymed.2012.07.009

[CIT0037] Zhao XN, Liang JL, Chen HB, Liang YE, Guo HZ, Su ZR, Li YC, Zeng HF, Zhang XJ. 2015. Anti-fatigue and antioxidant activity of the polysaccharides isolated from *Millettiae speciosae* Champ. Leguminosae. Nutrients. 7(10):8657–8669. 26506375 10.3390/nu7105422 PMC4632442

[CIT0038] Zhao Y, Shi Y, Yang H, Mao L. 2016. Extraction of *Angelica sinensis* polysaccharides using ultrasound-assisted way and its bioactivity. Int J Biol Macromol. 88:44–50. 26845475 10.1016/j.ijbiomac.2016.01.113

[CIT0039] Zhou X, Seto SW, Chang D, Kiat H, Razmovski-Naumovski V, Chan K, Bensoussan A. 2016. Synergistic effects of Chinese herbal medicine: a comprehensive review of methodology and current research. Front Pharmacol. 7:201. 27462269 10.3389/fphar.2016.00201 PMC4940614

